# A High Resolution On-Chip Delay Sensor with Low Supply-Voltage Sensitivity for High-Performance Electronic Systems

**DOI:** 10.3390/s150204408

**Published:** 2015-02-13

**Authors:** Duo Sheng, Hsiu-Fan Lai, Sheng-Min Chan, Min-Rong Hong

**Affiliations:** Department of Electrical Engineering, Fu Jen Catholic University, Taipei 24205, Taiwan; E-Mails: zero7260@hotmail.com (H.-F.L.); a2553858@hotmail.com (S.-M.C.); perp8825@hotmail.com (M.-R.H.)

**Keywords:** delay sensor, voltage variations, delay measurement, VLSI, all digital

## Abstract

An all-digital on-chip delay sensor (OCDS) circuit with high delay-measurement resolution and low supply-voltage sensitivity for efficient detection and diagnosis in high-performance electronic system applications is presented. Based on the proposed delay measurement scheme, the quantization resolution of the proposed OCDS can be reduced to several picoseconds. Additionally, the proposed cascade-stage delay measurement circuit can enhance immunity to supply-voltage variations of the delay measurement resolution without extra self-biasing or calibration circuits. Simulation results show that the delay measurement resolution can be improved to 1.2 ps; the average delay resolution variation is 0.55% with supply-voltage variations of ±10%. Moreover, the proposed delay sensor can be implemented in an all-digital manner, making it very suitable for high-performance electronic system applications as well as system-level integration.

## 1. Introduction

As semiconductor device size decreases, and the performance requirements of electronic products increase, the operating frequencies of advanced electronic systems can exceed several gigahertz, leading to narrower timing margins in the digital circuit. In the high-level integration systems, including system-on-chip (SoC), system-in-package (SiP), and 3D IC, the delay uncertainties, such as propagation delay mismatching, clock skew and jitter, degrade overall system performance and increase design efforts to meet the timing constraints; thus, these uncertainties cannot be ignored—particularly when small timing margins exist [1–5]. Furthermore, the timing-related defects that are caused by manufacturing-process-related problems, such as resistive opens and shorts, device mismatches, *etc.*, become serious problems [1,3,6]. Besides, non-ideal effects, such as negative bias temperature instability (NBTI), hot career injection (HCI), and electromigration (EM), will induce serious reliability issues after shipment [6,7]. Consequently, to ensure the functionality and performance of high-performing systems, it is necessary to develop a technology that measures either the delay in the critical path or timing uncertainty in the circuit [1–8].

Traditionally, delays can characterized by the off-chip method. However, because the accuracy of off-chip delay measurement is dominated by parasitic capacitance, probe resistance, and transmission line impedance, it is not suitable for internal nodes whose required timing resolution is at the picosecond level as the timing margin narrows. Besides, the increasing complexities of electronic systems limit the accessibility of internal circuit modules [2,5]. Thus, an on-chip delay sensor (OCDS) that can provide debugging for internal chip performance failure and delay diagnosis for high-frequency system applications is necessary. Moreover, at-speed delay measurements can provide the information required to improve overall performance and reduce the power consumption of equipment with dynamic voltage and frequency scaling (DVFS) schemes [8,9].

**Figure 1.**
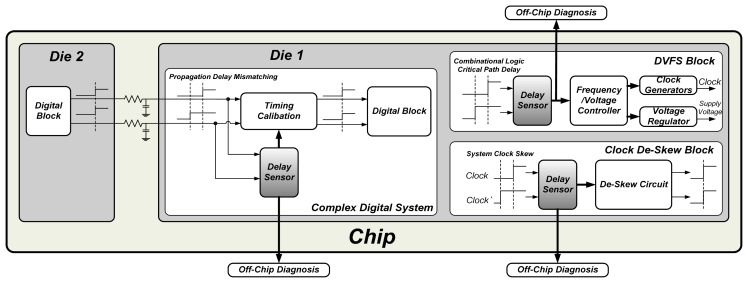
The scope of OCDS applications.

The scope of OCDS applications is illustrated in Figure 1. For example, the OCDS can detect the propagation delay mismatching between dies, and provide the sensing information for timing calibration or off-chip diagnosis. In the DVFS system, an OCDS circuit can measure the specified timing-critical path delay in a digital block and provide such delay information to the frequency/voltage controller. Based on the measured delay provided by the OCDS circuit, the operating frequency and supply voltage of the chip can be adjusted to improve overall performance and reduce power consumption. In addition to the timing-critical path delay measurement, the OCDS module can measure the clock skew between different clocks and output the measured data for clock de-skew circuit or diagnosis in an off-chip tester machine. Generally, because the considered transmission path and clock signal can be specified during the design phase, the number and location of the OCDS modules can be determined before chip fabrication.

Different approaches have been developed to implement an OCDS. The time-to-digital converter (TDC) can convert the measured delay to digital code. The design concept of TDC is very straightforward; however, its quantization resolution is not sufficient for advance system applications [10,11]. In contrast to TDC, measurement circuits based on a Vernier delay line (VDL) can achieve high delay resolution. However, such circuits have large hardware costs, and their delay resolutions are sensitive to supply-voltage variations [5,12]. A path-based ring oscillator structure that converts the target path's delay to the oscillation period has been proposed [13]. Although this architecture can be integrated into current design-for-testability (DFT) flow, its resolution is restricted by the oscillation period. To improve this delay resolution, an analogously tunable delay line, whose delay can be controlled with an analogous voltage, has been proposed [14]. This design can provide high delay resolution; however, it requires an additional high-quality digital-to-analog (DAC) circuit, which increases hardware costs and design effort. A differential delay line pair structure that provides high delay resolution has been proposed [15]. However, the detection range is insufficient for die-to-die timing calibration.

**Figure 2.**
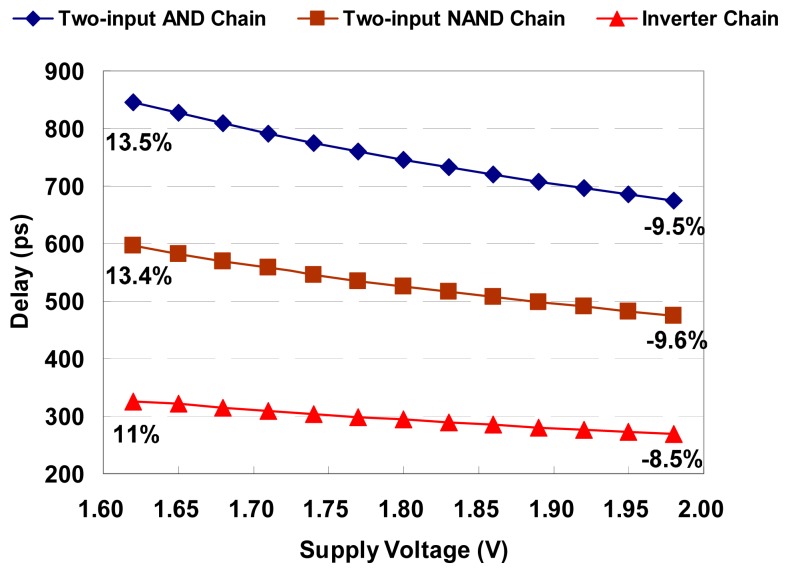
The delay spread of a delay line with supply-voltage variations.

The most important criterion of OCDS is its quantization resolution and timing quantization stability, both of which determine the accuracy and quality of its delay measurement. Basically, the digital approach utilizes the delay cell's delay, such as the buffer or AND gate, as the overall quantization resolution. Unfortunately, in the complementary metal-oxide-semiconductor (CMOS) process, gate delay is strongly affected by supply-voltage variations. Figure 2 shows the delay spread of a delay line composed of 10 basic logic gates connected in a series in a 0.18 μm CMOS process. For example, in the two-input AND delay chain, the delay varies widely from 675 to 846.4 ps with ±10% supply-voltage variations, which means the percentages of the delay variation value are −9.5% and 13.5%. In complex digital systems, the supply voltage of OCDS varies easily due to nearby circuit influences or other non-ideal effects; therefore, the delay resolution of the delay line is unpredictable and unstable. Consequently, the measurement accuracy of OCDS is significantly degraded by supply-voltage variations, and OCDS with low immunity to supply variations is unsuitable for in-demand high-precision applications.

In this paper, an all-digital, high-resolution OCDS circuit with high immunity to supply-voltage variations is proposed for high-performance electronic system applications. The proposed OCDS uses a novel, closed-loop delay measurement to provide high delay-measurement resolution. Furthermore, the proposed OCDS employs a cascade-stage delay measurement circuit, including a coarse delay measurement stage (CDMS) and two fine delay measurement stages (FDMS), and a high-sensitivity phase detector (PD) to enhance the immunity of the overall delay resolution to supply-voltage variations. Moreover, the complete design of the proposed OCDS can be implemented in an all-digital manner, making its integration into a digital system easy.

This paper is organized as follows: Section 2 describes the proposed delay measurement scheme and hardware architecture. Section 3 focuses on the key module circuit design of OCDS, including CDMS, FDMS, and PD. In Section 4, the implementation and simulation results of the proposed design are presented. Finally, the conclusions are addressed in Section 5.

## 2. Sensor Overview

**Figure 3.**
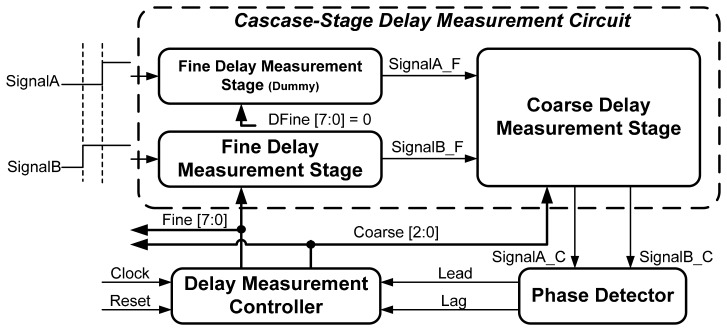
The proposed all-digital OCDS architecture.

Figure 3 illustrates the proposed all-digital OCDS architecture, which comprises a cascade-stage delay measurement circuit, a PD, and a delay measurement controller. The proposed cascade-stage delay measurement circuit comprises a CDMS, a FDMS, and a dummy fine delay measurement stage. The coarse and fine delay measurement stages form the controllable delay line with different tunable delay steps. The CDMS has coarse quantization resolution, and the delay is controlled by the coarse control code *Coarse[2:0]*. In contrast to the CDMS, the FDMS has a smaller controllable delay to increase the overall delay measurement resolution, and the delay of the fine measurement stage is controlled by the fine control code *Fine[7:0]*. To maintain intrinsic delay matching during delay measurement, the dummy FDMS whose control code *(DFine[7:0]*) is fixed to zero is added. If the timing difference between *SignalA* and *SignalB* requires measurement, *SignalA* and *SignalB* are sent to cascade-stage delay measurement as inputs of the dummy fine measurement stage and fine measurement stage, respectively.

After *SignalA* and *SignalB* propagate through the fine delay measurement stages, the first delayed version signals (*SignalA_F* and *SignalB_F*) are sent to the CDMS, which generates the second delayed version signals (*SignalA_C* and *SignalB_C*). Based on the phase polarity between *SignalA_C* and *SignalB_C*, the phase detector generates the phase comparison results *Lead* and *Lag*. According to the phase comparison results, the measurement controller changes digital control codes to tune the CDMS and FDMS delays until the phases of *SignalA_C* and *SignalB_C* align.

**Figure 4.**
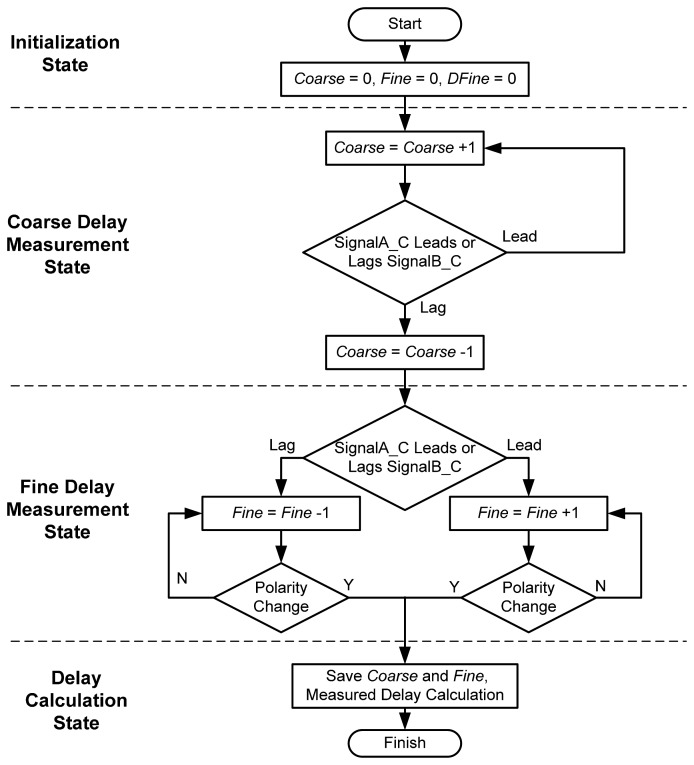
The flowchart of the proposed OCDS.

The flowchart of the proposed OCDS is shown in Figure 4. The entire delay measurement process is divided into coarse and fine measurement states. In the beginning, two control codes, *Coarse* and *Fine*, are initialized to zero. Subsequently, the phase detector asserts a digital signal, either *Lead* or *Lag*, based on the phase polarity of the *SignalA_C* rising edge to the *SignalB_C* rising edge. We assume that the positive edge of *SignalA* leads the positive edge of *SignalB*; therefore, in the beginning, the positive edge of *SignalA_C* will lead the positive edge of *SignalB_C*. Subsequently, the phase detector asserts *Lead*, and the delay measurement controller increases *Coarse* by one to enlarge the propagation delay from *SignalB_F* to *SignalB_C* until the positive edge of *SignalA_C* lags behind that of *SignalB_C*. Once the phase detector senses a change in the phase polarity of *SignalA_C* relative to *SignalB_C*, the delay measurement controller decreases *Coarse* by one, and the coarse measurement state is complete.

The operation of the fine delay measurement state and coarse delay measurement state are similar. In the fine delay measurement state, the delay measurement controller determines *Fine* to change the propagation delay from *SignalB* to *SignalB_F*. If the positive edge of *SignalA_C* leads that of *SignalB_C*, the measurement controller increases *Fine* by one to enlarge the propagation delay from *SignalB* to *SignalB_F*. Conversely, if the phase detector asserts *Lag*, the delay measurement controller decreases *Fine* by one to reduce the propagation delay from *SignalB* to *SignalB_F*. Phase comparison continues until the phase detector senses a change in the phase polarity of *SignalA_C* relative to *SignalB_C*. At this point, *Coarse* and *Fine* will be saved, and the delay measurement is complete.

Because the positive edges of *SignalA_C* and *SignalB_C* will be aligned after the delay measurement is complete, the delay between *SignalA* and *SignalB* is equal to the propagation delay difference between *SignalA* to *SignalA_C* and *SignalB* to *SignalB_C*, which can be formulated as:

(1) $$T_{B}=T_{M}+T_{A}$$

*T_A_* and *T_B_* are the propagation delays from *SignalA* to *SignalA_C* and *SignalB* to *SignalB_C*, respectively. *T_M_* is the delay between *SignalA* and *SignalB*. The propagation delay from *SignalA* to *SignalA_C* (*T_A_*) can be divided into two parts: from *SignalA* to *SignalA_F* (*T_AF_*) and from *SignalA_F* to *SignalA_C* (*T_AC_*). *T_B_* is similar to *T_A_*; it can be divided into two parts: from *SignalB* to *SignalB_F* (*T_BF_*) and from *SignalB_F* to *SignalB_C* (*T_BC_*). *T_A_* and *T_B_* can be expressed by the intrinsic delay of the measurement stage, the delay step of the cascade-stage delay measurement circuit, and the coarse and fine control codes. Thus, they can be formulated as:

(2) $$\begin{array}{l} T_{A}=T_{AF}+T_{AC}=\left(T_{IF}+\left(\text{DFine}\times \Delta T_{F}\right)\right)+T_{IC}\\ T_{B}=T_{BF}+T_{BC}=\left(T_{IF}+\left(\text{Fine}\times \Delta T_{F}\right)\right)+\left(T_{IC}+\left(\text{Coarse}\times \Delta T_{C}\right)\right) \end{array}$$

**Figure 5.**
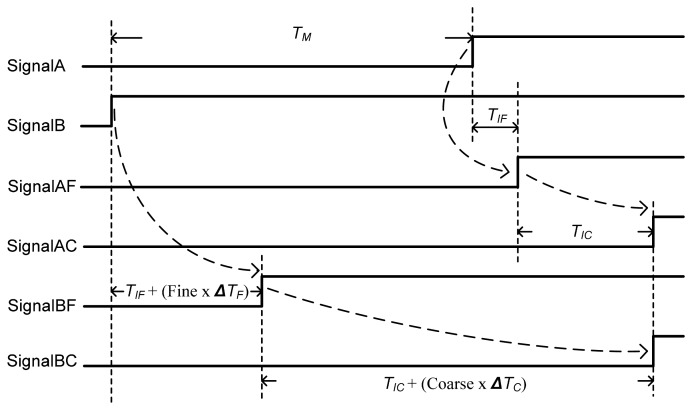
The signal timing in the proposed OCDS.

Ä*T_C_* and Ä*T_F_* are the quantization resolution (delay step) of CDMS and FDMS, respectively. *T_IC_* and *T_IF_* are the intrinsic delay of CDMS and FDMS, respectively. According to Equations (1) and (2), the delay between *SignalA* and *SignalB* (*T_M_*) can be quantized by the FDMS's finest delay step, and the measured delay can be calculated by the quantization resolution, *Coarse*, and *Fine*, as shown in Figure 5. It can be formulated as:

(3) $$T_{M}=T_{B}-T_{A}=\left(\text{Coarse}\times \Delta T_{C}\right)+\left(\text{Fine}\times \Delta T_{F}\right)$$

(4) $${\text{if}}^{\Delta T_{C}=N\times \Delta T_{F}},T_{M}=\left(\text{Coarse}\times N+\text{Fine}\right)\times \Delta T_{F}$$

The relationship between the delay steps of CDMS and those of FDMS is stable in the proposed cascade-stage delay measurement circuit. Moreover, according to Equation (4), the quantization resolution of the OCDS circuit is equal to ÄT_F_. Thus, if ÄT_F_ is insensitive to supply-voltage variations, the same measured delay (T_M_) will have the same quantization results (Coarse and Fine) when supply-voltage variations are present. As a result, the delay measurement results of the OCDS will not be affected by supply-voltage variations. The rest of this paper describes how to implement a high quantization resolution OCDS whose finest delay step is insensitive to supply-voltage variations.

## 3. Circuit Implementation

### 3.1. Coarse Delay Measurement Stage

**Figure 6.**
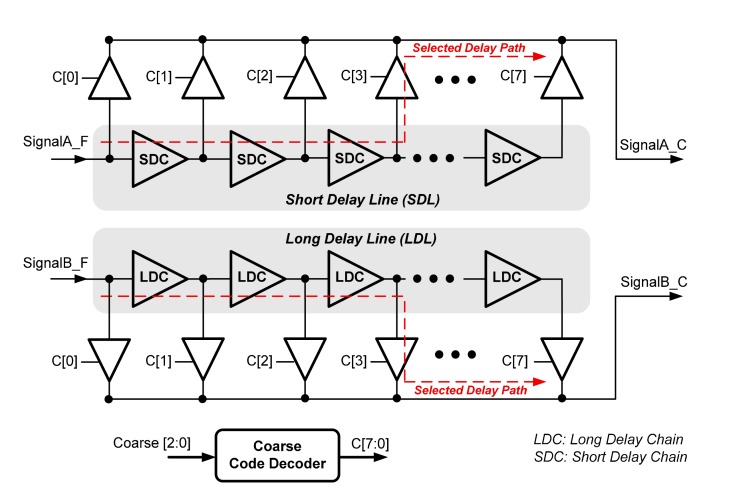
The circuit diagrams of the proposed CDMS.

The proposed CDMS is composed of a long delay line, short delay line, coarse code decoder, and sixteen tri-state buffers, as shown in Figure 6. The long and short delay line comprise seven long delay chains (LDC) and seven short delay chains (SDC), respectively. The proposed coarse code decoder receives *Coarse[2:0]* from the delay measurement controller and generates path selection signals *C[7:0]* to the tri-state buffers. The coarse code decoder converts binary code to one-shot code. As *SignalA_F* propagates through short delay line, it can provide eight different delay values by selecting different delay paths organized by these seven SDCs. Similar to *SignalA_F, SignalB_F* propagates through long delay line and a turned-on tri-state buffer (determined by *C[7:0])* to *SignalB_C*. Based on the CDMS's circuit structure, the propagation delays of *SignalA_F* to *SignalA_C* (*T_AC_*) and *SignalB_F* to *SignalB_C* (*T_BC_*) can be formulated as:

(5) $$\begin{array}{l} T_{AC}=\left(\text{Coarse}\times T_{\text{SDC}}\right)+T_{\text{TBUF}}\\ T_{BC}=\left(\text{Coarse}\times T_{\text{LDC}}\right)+T_{\text{TBUF}} \end{array}$$

*T_LDC_* and *T_SDC_* are the LDC and SDC delays, respectively. *T_TBUF_* is the delay of a tri-state buffer. Therefore, the delay difference between these two propagation delays is:

(6) $$T_{BC}-T_{AC}=\text{Coarse}\times \left(T_{\text{LDC}}-T_{\text{SDC}}\right)=\text{Coarse}\times \Delta T_{C}$$

According to Equation (6), the quantization resolution (delay step) of CDMS is equal to the delay difference between the LDC and SDC, and the measured delay can be roughly quantized by Ä*T_C_*.

### 3.2. Delay Cell

In the proposed OCDS design, the most important design consideration is how to maintain measurement accuracy when supply-voltage variations exist. Because measurement accuracy is determined by the stability of the delay cell in cascade-stage delay measurement circuit, it is important to enhance the immunity that CDMS and FDMS delay quantization resolutions have to supply-voltage variations. According to Equation (6), the delay quantization resolution of CDMS is determined by the delay difference between the LDC and SDC. Thus, if the delay difference between the LDC and SDC is insensitive to supply-voltage variations, the stability and accuracy of the CDMS's measurement can be ensured.

When the supply voltage change, the propagation delay of the LDC and SDC can be formulated as:

(7) $$T_{\text{LDC}1}=T_{\text{LDC}0}+\Delta T_{\text{LDC}},T_{\text{SDC}1}=T_{\text{SDC}0}+\Delta T_{\text{SDC}}$$

(8) $${\text{if}}^{\Delta T_{\text{LDC}}=\Delta T_{\text{LDC}}}{,}^{T_{\text{LDC}}-T_{\text{SDC}}=T_{\text{LDC}0}-T_{\text{SDC}0}}$$

T_LDC1_ and T_SDC1_ are the propagation delays the LDC and SDC, respectively, after supply changes. T_LDC0_ and T_SDC0_ are the propagation delays the LDC and SDC, respectively, before supply changes. ΔT_LDC_ and ΔT_SDC_ are the delay variations of the LDC and SDC, respectively, caused by supply changes. If ΔT_LDC_ is equal to ΔT_SDC_, the delay difference between the LDC and SDC (ΔT_C_) will maintain a constant value under different supply voltages, as shown in Equations (7) and (8).

**Figure 7.**
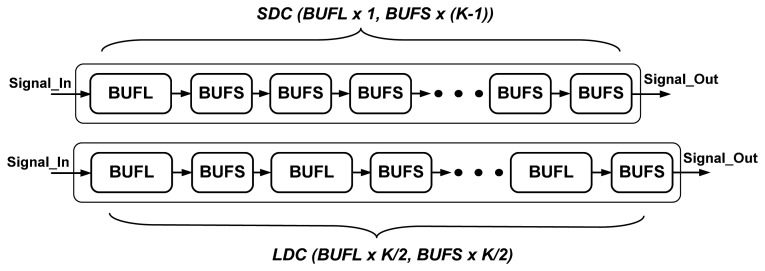
The circuit diagrams of the proposed LDC and SDC.

The circuit diagrams of the proposed LDC and SDC are shown in Figure 7. The proposed LDC comprises *K*/*2* large (BUFL) and *K*/*2* small buffers (BUFS). The proposed SDC comprises one large (BUFL) and *K* − *1* small buffers (BUFS). The large and small buffers determine not only the LDC and SDC overall delays, but also the delay variations caused by supply changes. The large and small buffers are rise/fall times balance buffers and have the same circuit structure; the only difference between them is the channel width and length of each MOS, which leads to different obtained delays. If the LDC and SDC use the same buffer, both delay variations caused by supply changes will be the same. However, these two delay cells also have the same propagation delay, which is not suitable for CDMS requirements.

Thus, at first, the LDC and SDC comprise *K* large and *K* small buffers, respectively. In order to reduce the delay variation difference between the LDC and SDC caused by supply changes, *K*/*2* large buffers are replaced by *K*/*2* small buffers in the LDC. Furthermore, *K*/*2* large and *K*/*2* small buffers have interleaved placement. The first small buffer in the SDC is replaced by a large buffer so that LDC and SDC have the same driving capability. The LDC and SDC delays need to be characterized under different supply voltages in the HSPICE simulation, and maintaining the delay difference between the LDC and SDC requires a constant value under different supply voltages, as shown in Equation (8).

**Figure 8.**
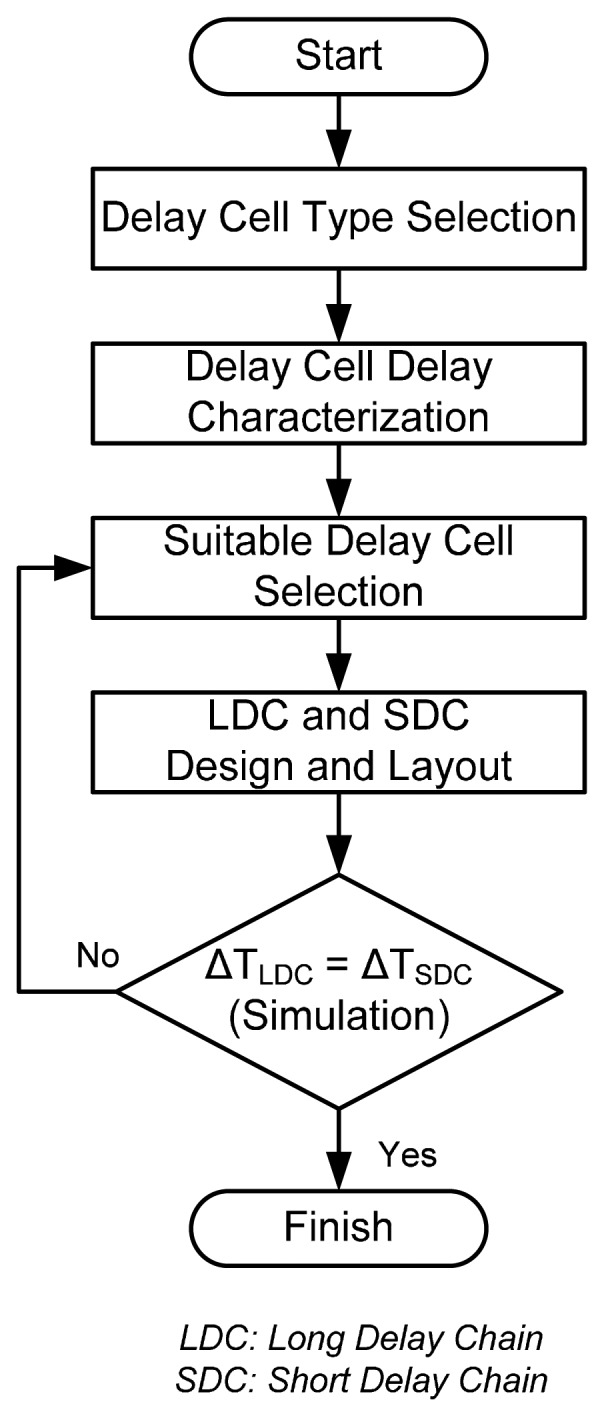
The flowchart of the proposed design procedure.

The implementation of the BUFL and BUFS, as used in the proposed long delay and short chains, is a challenge in our design. We propose a systematic design flow to determine the transistor sizes and the layout style of the buffer to improve performance. The flowchart of the proposed design flow is shown in Figure 8. In order to reduce the design and layout complexity, we use the delay cells from the standard cell library. In the beginning, one type of delay cell in the standard cell library is selected. After the layout, the delays of all the buffers different driving capability are characterized as a function of supply voltage variation. Based on the delay characterization results, the suitable buffers can be selected for the long and short delay chains. Finally, if Δ*T_LDC_* is equal to Δ*T_SDC_*, Δ*T_C_* will maintain a constant value under different supply voltages. If not, another buffer for the long and short delay chains must be selected until our design target is achieved.

### 3.3. Fine Delay Measurement Stage

**Figure 9.**
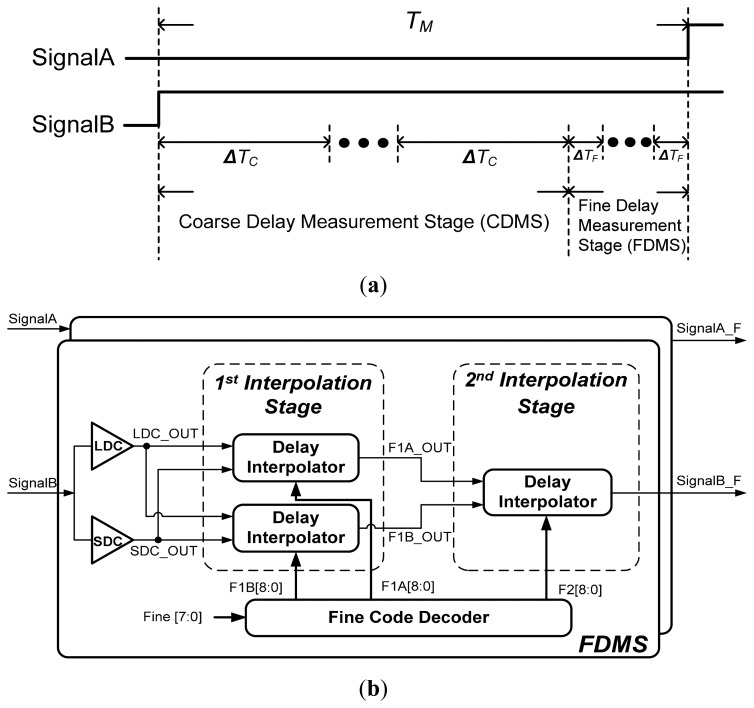

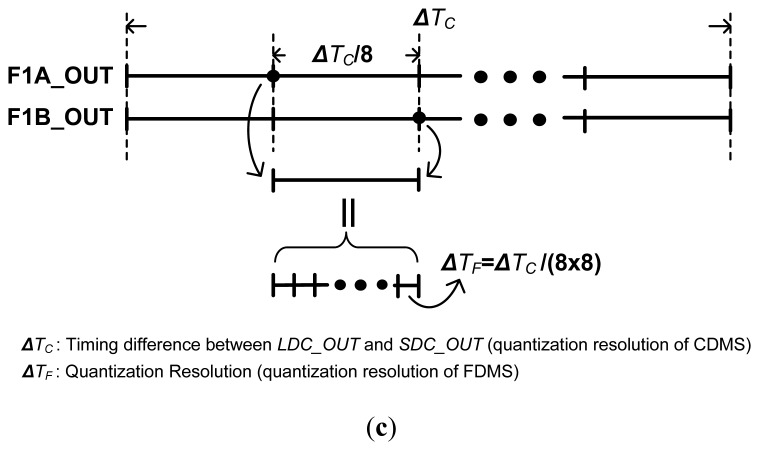
(**a**) The operation concept of the proposed cascade-stage delay measurement; (**b**) The circuit diagrams of the proposed FDMS; (**c**) The operation concept of two-stage delay interpolation.

Although the delay quantization resolution of CDMS can maintain a constant value when the supply voltage changes, it is still insufficient for high-performance system applications. Thus, an FDMS is added to the proposed cascade-stage delay measurement circuit to improve the overall delay quantization resolution from one gate delay to several picoseconds. The operation concept of the proposed cascade-stage delay measurement circuit is illustrated in Figure 9a. The delay between *SignalA* and *SignalB* will be measured by the CDMS and FDMS. After the CDMS measures, the time residue will be measured by FDMS to further improve the measurement quantization. In order to maintain immunity to supply-voltage variations, the ratio of delay quantization resolution in the CDMS to that in FDMS has to maintain a constant value, and the delay quantization resolution of FDMS should also have a high immunity to supply-voltage variations.

The proposed FDMS is composed of an LDC, SDC, first interpolation stage, second interpolation stage, and fine code decode, as shown in Figure 9b. The proposed FDMS employs a two-stage delay interpolation structure—the inputs that *SignalA* and *SignalB* send to the corresponding FDMS, respectively—to improve the delay quantization resolutions. Because the propagation path of two input signals is the same, they have the same intrinsic delay. If the positive edge of *SignalA* leads the positive edge of *SignalB*, the fine control code for FDMS that *SignalA* passed will be set to zero *(DFine[7:0])*. Meanwhile, the fine control code for FDMS that *SignalB* passed will be increased to reduce the phase difference between *SignalA_F* and *SignalB_F*.

Because the delay interpolator can divide the delay between two input signals into equal parts, if the timing difference between LDC_OUT and SDC_OUT is Δ*T_C_* and the interpolation step of the first and second interpolation stages are Q and R, respectively, the overall delay resolution of the OCDS will be Δ*T_C_*/(Q × R), as shown in Figure 8c. According to the design specification, the design parameters are determined as follows: Q = 8 and R = 8. The first interpolation stage receives LDC_OUT and SDC_OUT from the LDC and SDC, respectively, and then interpolates those signals to generate two signals (F1A_OUT and F1B_OUT). The delays of the first and second interpolation stages are controlled by {*F1A[8:0], F1B[8:0]*} and *F2[8:0]*, respectively, which are generated by the code decoders.

**Figure 10.**
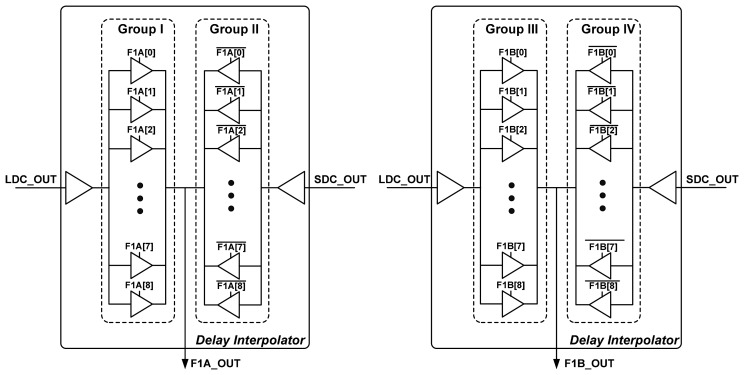
The architecture of the proposed first interpolation stage.

Figure 10 illustrates the architecture of the proposed first interpolation stage, which comprises two delay interpolators [16]. Each delay interpolator comprises two driving groups: Group I–IV. The proposed first interpolation stage generates two outputs (F1A_OUT and F1B_OUT) whose timing difference is one 1st tuning delay step. Table 1 lists the combinations of the four control group codes. The single delay step of the first interpolation stage has been “extracted” by the 1st tuning control code; the second interpolation stage receives these two outputs and further improves delay resolution by the delay interpolator.

Because the delay difference between the LDC and SDC (Δ*T_C_*) and the interpolation step of two interpolation stages (8, 8) are not affected by supply variation, the overall delay quantization resolution of the proposed OCDS (Δ*T_C_*/(8 × 8)) is insensitive to supply-voltage variations. As a result, the proposed OCDS can not only achieve high delay resolution, it can also have a high immunity to supply-voltage variations.

### 3.4. Phase Detector

In order to improve measurement accuracy, the proposed OCDS employs a sense-amplifier-based PD to ensure that the overall delay resolution will not be reduced by the PD's dead zone [17]. Based on simulation results, the sense-amplifier-based PD can detect a phase error greater than 1 ps; thus, it is very suitable for the proposed OCDS design.

## 4. Simulation Results

**Figure 11.**
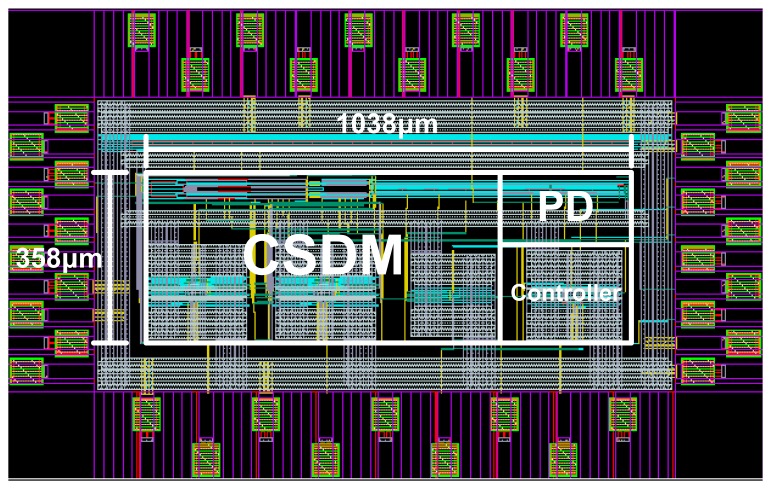
Layout of the proposed OCDS.

The proposed all-digital OCDS is implemented in a 0.18 μm 1P6M CMOS process. The design parameters of the proposed cascade-stage delay measurement circuit are determined on the basis of requested delay measurement resolution that is based on our application. The layout of the OCDS is shown in Figure 11; its area is 1038 μm × 358 μm.

**Figure 12.**
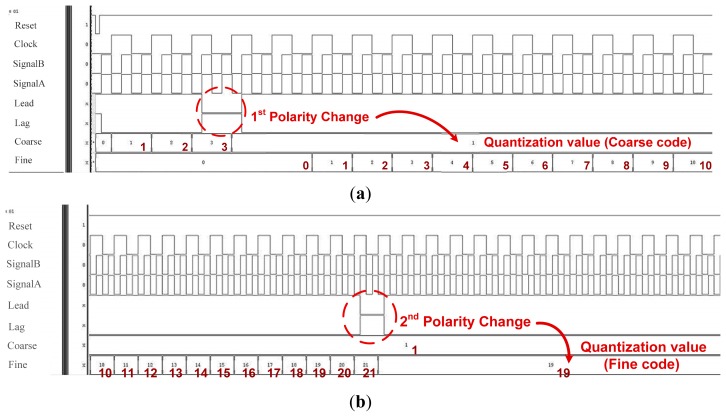
The quantization process of (**a**) coarse and (**b**) fine delay measurement.

The proposed OCDS is designed and implemented using an all-digital design flow; thus, the proposed architecture and algorithm are modeled in the hardware description language (HDL) and the functionally is verified using an NC-Verilog simulator. Figure 12a,b show the quantization process of the coarse and fine delay measurement, respectively. The simulation results of the proposed OCDS scheme show that the target measured delay is converted to the quantization value. The entire delay measurement process is divided into coarse and fine measurement states. In the beginning, two control codes, *Coarse* and *Fine*, are initialized to zero. If the positive edge of *SignalA* leads the positive edge of *SignalB*, the delay measurement controller increases *Coarse* by one to enlarge the propagation delay from *SignalB_F* to *SignalB_C* until the phase detector senses a change in the phase polarity. The delay measurement controller decreases *Coarse* by one, and the coarse measurement state is complete. In the fine delay measurement state, if the positive edge of *SignalA* leads the positive edge of *SignalB*, the measurement controller increases *Fine* by one to enlarge the propagation delay from *SignalB* to *SignalB_F*. Phase comparison continues until the phase detector senses a change in the phase polarity. At this point, *Coarse* and *Fine* will be saved, and the delay measurement is complete.

In order to verify the performance of the proposed OCDS precisely, post-layout simulation of the timing-related part uses HSPICE for enhanced accuracy. To test the delay measurement characteristic of the proposed OCDS, input time intervals of 0–600 ps with a 25 ps step are applied to the system. The simulation shows the measurement characteristic with ±10% supply-voltage variations (from 1.62 V to 1.98 V). The average measurement resolution is 1.2 ps. The measured delay can be obtained from the output digital code by measurement resolution. The output digital code comprises *Coarse* and *Fine*. Because the delay quantization resolution of *Coarse* is 64 times that of *Fine*, *Coarse* needs to multiply by 64, combine with *Fine*, and then convert to decimal-format digital code. For example, if *Coarse* is 2 and *Fine* is 32, the output digital code is equal to 160 (2 × 64 + 32) and the measured delay is 192 ps (160 × 1.2 ps).

**Figure 13.**
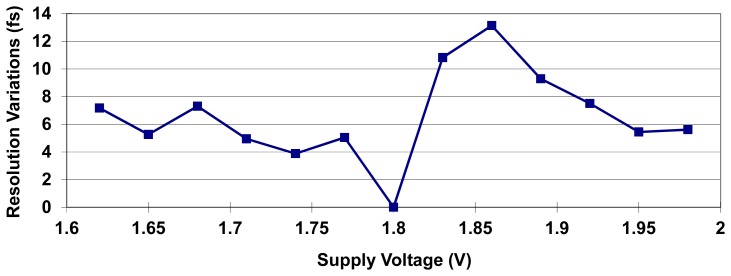
Quantization resolution deviation over a supply-voltage range of 1.8 V ± 10%.

**Figure 14.**
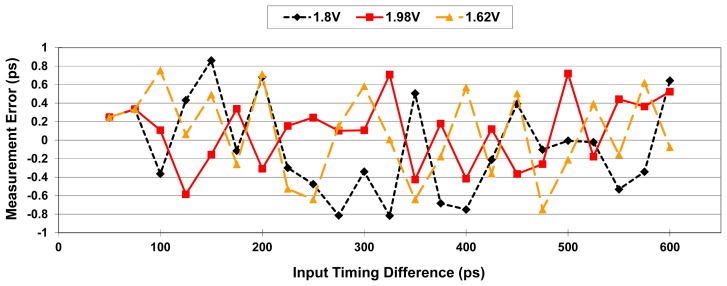
The measurement error of the proposed OCDS.

**Figure 15.**
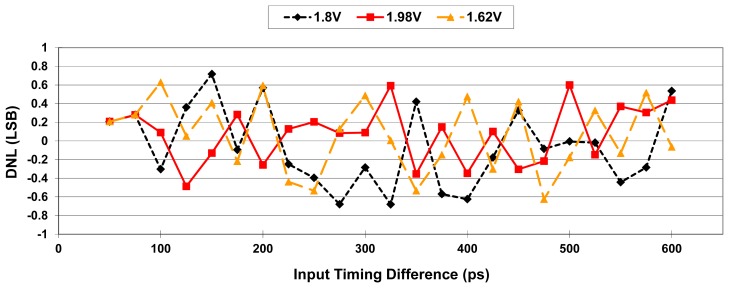
DNL of the proposed OCDS.

To evaluate supply-voltage sensitivity, the resolution deviation of the system is determined by simulation over a supply-voltage range of 1.8 V ± 10%. Figure 13 shows the simulation results for supply voltages ranging from 1.62 V to 1.98 V in 0.05 V steps, presenting an average 0.55% and maximum 1.1% resolution deviation over the specified supply voltage range. The corresponding measurement error and differential nonlinearity (DNL) are also calculated to evaluate the linearity of the system, as shown in Figures 14 and 15, respectively. DNL errors of less than 0.72 LSB, 0.6 LSB, and 0.63 LSB with supply voltages of 1.8 V, 1.98 V, and 1.62 V, respectively, are obtained.

Table 2 lists comparison results with state-of-the-art OCDSs for electronic system applications. The proposed OCDS can achieve high measurement resolution and low power consumption. Furthermore, the measurement resolution of the proposed OCDS has high immunity to supply-voltage variations (0.22 ps/V). Although [14] has a better measurement resolution, it needs an extra 16-bit DAC to provide fine measurement resolution, which leads it to consume a lot of power and occupy a large chip area. Because the proposed OCDS is designed for short-time-interval measurement in high-performance electronic system applications, the detection range is shorter than that of prior works. For long-time-interval measurement, the input range can be extended by adding more the LDCs and SDCs to the CDMS. Additionally, as the proposed DCO can be implemented with an all-digital design, it is more suitable for system integration than prior works. As a result, the proposed OCDS has the benefits of better measurement resolution, power consumption, and voltage sensitivity.

## 5. Conclusions

In this paper, we have proposed an all-digital, high-resolution, and low-supply-sensitive ODCM design for advanced electronic system applications. Based on the proposed ODCM scheme, the input delay can be converted to a digital quantization value with fine delay measurement resolution. The proposed cascade-stage delay measurement circuit can not only enhance the immunity to supply-voltage variations of delay measurement resolution without an extra self-biasing or calibration circuit, it can also achieve high delay resolution. The proposed design was implemented using the 0.18 μm 1P6M CMOS process, and post-layout simulations are performed to confirm the validity of the design approach. Simulation results show that delay measurement resolution can be improved to 1.2 ps and that the average delay resolution variation is 0.55% with ±10% supply-voltage variations. Furthermore, the proposed design can be implemented in an all-digital design manner, making it very suitable for high-performance electronic system applications as well as system-level integration.

## Figures and Tables

**Table 1.** Timing control of first interpolation stage.

**Control Code F1A[8:0]**	**Control Code F1B[8:0]**	**F1A_OUT Timing**	**F1B_OUT Timing**
000000001	000000000	TSD + S	TSD
000000011	000000001	TSD + 2S	TSD + S
000000111	000000011	TSD + 3S	TSD + 2S
000001111	000000111	TSD + 4S	TSD + 3S
000011111	000001111	TSD + 5S	TSD + 4S
000111111	000011111	TSD + 6S	TSD + 5S
001111111	000111111	TSD + 7S	TSD + 6S
011111111	001111111	TSD + 8S	TSD + 7S
111111111	011111111	TLD	TSD + 8S

TLD: Timing of LDC_OUT, TSD: Timing of SDC_OUT, S: Delay step of first interpolation stage (*ΔT_C_*/8).

**Table 2.** Performance Comparisons.

**Performance Indices**	**Proposed Design**	**TIM'09 [11]**	**TVLSI'12 [3]**	**EL'12 [14]**	**JSSC'06 [18]**
Process	0.18 μm CMOS	0.18 μm CMOS	0.18 μm CMOS	90 nm CMOS	0.35 μm CMOS
Supply Voltage (V)	1.8	1.8	NA	1	3.3
LSB Resolution	1.2 ps	14.4 ps	6.62 ps	28.1 fs (with 16-bit DAC)	12.2 ps
Resolution Variations with Supply Voltage	0.22 ps/V	10 ps/V	NA	NA	NA
Measurement Range	600 ps	460 ps	3295 ps	108.8 ps	202 μs
Power Consumption	1.8 mW	3.6 mW	NA	NA	40 mW
